# The Influence of Obesity and Weight Loss on the Bioregulation of Innate/Inflammatory Responses: Macrophages and Immunometabolism

**DOI:** 10.3390/nu14030612

**Published:** 2022-01-30

**Authors:** Isabel Gálvez, María Carmen Navarro, Leticia Martín-Cordero, Eduardo Otero, María Dolores Hinchado, Eduardo Ortega

**Affiliations:** 1 Immunophyisiology Research Group, Instituto Universitario de Investigación Biosanitaria de Extremadura (INUBE), 06071 Badajoz, Spain; igalvez@unex.es (I.G.); cnavarropz@unex.es (M.C.N.); leticiamartin@unex.es (L.M.-C.); eoteroc@unex.es (E.O.); mhinsan@unex.es (M.D.H.); 2 Immunophysiology Research Group, Nursing Department, Faculty of Medicine and Health Sciences, University of Extremadura, 06071 Badajoz, Spain; 3 Immunophysiology Research Group, Physiology Department, Faculty of Sciences, University of Extremadura, 06071 Badajoz, Spain; 4 Immunophysiology Research Group, Nursing Department, University Center of Plasencia, University of Extremadura, 10600 Plasencia, Spain

**Keywords:** obesity, inflammation, innate response, macrophages, immunometabolism, weight loss

## Abstract

Obesity is characterized by low-grade inflammation and more susceptibility to infection, particularly viral infections, as clearly demonstrated in COVID-19. In this context, immunometabolism and metabolic flexibility of macrophages play an important role. Since inflammation is an inherent part of the innate response, strategies for decreasing the inflammatory response must avoid immunocompromise the innate defenses against pathogen challenges. The concept “bioregulation of inflammatory/innate responses” was coined in the context of the effects of exercise on these responses, implying a reduction in excessive inflammatory response, together with the preservation or stimulation of the innate response, with good transitions between pro- and anti-inflammatory macrophages adapted to each individual’s inflammatory set-point in inflammatory diseases, particularly in obesity. The question now is whether these responses can be obtained in the context of weight loss by dietary interventions (low-fat diet or abandonment of the high-fat diet) in the absence of exercise, which can be especially relevant for obese individuals with difficulties exercising such as those suffering from persistent COVID-19. Results from recent studies are controversial and do not point to a clear anti-inflammatory effect of these dietary interventions, particularly in the adipose tissue. Further research focusing on the innate response is also necessary.

## 1. Introduction

### 1.1. Obesity and Immune System

Diet-induced obesity is a leading health concern globally, considering its associated healthcare costs, morbidity, and mortality [1]. This major global public health issue is commonly linked to a wide variety of disorders, particularly metabolic disorders such as insulin resistance, type 2 diabetes, and dyslipidemia [2], but also cancer, cardiovascular diseases, and respiratory and immune dysfunction [3]. In fact, metabolic syndrome has been identified as a complex metabolic disorder associated with obesity that involves risk factors for type 2 diabetes and arteriosclerosis, thus increasing the risk of cardiovascular events [4]. The close relations between endocrine/metabolic functions and the immune system can explain the impairment of the innate/inflammatory responses that take place in this pathology.

Thus, nutritional status can deeply affect immune system functions. Optimal nutritional and metabolic homeostasis is a crucial part of appropriate immune function and health status. When a chronic positive imbalance between energy intake and expenditure occurs, the consequence is an increase in body mass that can lead to obesity, which in turn can influence innate and adaptive immune responses. First, regarding defense capacity against pathogens, clinical and epidemiological evidence has shown that the incidence and severity of different types of infectious illnesses are higher in obese individuals compared to lean individuals, together with the presence of poor antibody responses to antigens in these subjects [5,6], for example in bacterial infections such as pneumococcal diseases or infections with *Listeria monocytogenes* or *Klebsiella pneumoniae* [6], viral infections such as dengue fever [7], parasitic diseases such as malaria [8], and fungal infections such as candidiasis [6]. Moreover, recently, obesity has been clearly associated with the higher prevalence, bad prognoses, and worse outcomes in COVID-19. Although the exact underlying mechanisms are not completely elucidated yet, the higher risk associated with obesity has been suggested to be due to the pro-inflammatory state, which may facilitate cytokine storms, to dysregulated immune responses, including alterations in leukocyte count, function, and distribution, and to the upregulation of angiotensin-converting enzyme 2 (ACE2) receptor [9]. These data indicate the presence of altered immune activation and suppressed ability to fight pathogens in obesity. In this context, the results from our laboratory showed that immune cells from obese animals present lower phagocytic and microbicide capacities than those from lean ones [10], together with dysregulation in the spontaneous release of pro-inflammatory cytokines and stress mediators [11,12,13] and an immune-neuroendocrine dysregulation involving the feedback mechanism between interleukin (IL)-6 and noradrenaline, all of which contributes to chronic low-grade inflammation in this pathology.

### 1.2. Obesity and Low-Grade Inflammation

Low-grade inflammation is a condition in which there are elevated systemic concentrations of tumor necrosis factor-alpha (TNF-α), IL-1β, IL-6, IL-1 receptor antagonist (IL-1ra), and C-reactive protein (CRP) [14]. Low-grade inflammation is a major characteristic of obesity and metabolic syndrome, and these inflammatory molecules play a key role in the pathophysiology of concomitant metabolic disorders, particularly insulin resistance [15]. The term “metainflammation”, or metabolically triggered inflammation, also refers to low-grade systemic inflammation due to its strong relationship with the development of cardio-metabolic diseases in obesity [16]. Furthermore, the neuroendocrine disorders present in obesity (noradrenergic dysfunction, elevated circulating levels of the stress mediators noradrenaline and 72-kd heat shock protein [Hsp72], defective regulation of the negative inflammatory/stress feedback loop involving noradrenaline) are linked to abnormalities in the innate immune and inflammatory responses and also contribute to obesity-related metabolic complications [17,18]. Circulating monocytes from obese mice and humans have also shown a pro-inflammatory phenotype and cytokine profile [19], and obesity-induced alterations in the noradrenergic modulation of this cell type were also demonstrated [20,21]. In this context, the concept of “bioregulation” is particularly relevant since it takes into account both the innate immune response and the inflammatory response: bioregulation is achieved when an excessive inflammatory response is reduced or prevented while preserving or stimulating the innate response. In this way, the reduction in the elevated inflammatory response is not accompanied by further suppression of the innate response in low-grade inflammation-related pathologies [22,23]. 

In addition to low-grade systemic inflammation, local inflammation is present in several metabolically active organs in this condition: liver, pancreas, skeletal muscle, and, most importantly, adipose tissue [15,24]. It is characterized by increased expression of inflammatory mediators and qualitative and quantitative changes in the leukocytes of metabolic tissues, including the hypothalamus, liver, and adipose tissue [25,26,27]. In fact, systemic inflammation can be considered both a reflection of local inflammatory responses and also a major contributor to the perpetuation of these local responses. However, the exact mechanisms concerning the onset and interactions of the altered local and systemic inflammatory responses in obesity are still not clear. In this context, most lines of evidence have focused on adipose tissue inflammation. In recent years, the understanding of the role of inflammation in metabolic tissues has become more accurate. It is now understood that the initial inflammatory response in the adipose tissue, possibly induced by hypoxia, adipocyte death, mechanical stress, or gut-derived antigens, is acute and protective. Only after a long-term period, inflammation becomes chronic and detrimental due to maladaptation [28].

### 1.3. Obesity and Adipose Tissue Inflammation

In the early 1990s, some studies showed the first pivotal observations of the inflammatory origin of obesity and diabetes. In 1993, Hotamisligil and colleagues [29] discovered that adipocytes, in rodents, secreted the pro-inflammatory cytokine TNF-α. Although it was unknown to them, this discovery later shaped the future of obesity research worldwide, constituting the first molecular link between obesity and inflammation. In the mid-1990s, it was demonstrated that inflammatory cytokines such as TNF-α and IL-6 were higher in obese adipose tissue compared to that from lean individuals and that this event could induce insulin resistance in cells such as adipocytes, myocytes, and hepatocytes, beginning new lines of research into the immune regulation of metabolic processes [30,31]. Following the discovery of cytokine-like leptin by Zhang and colleagues in 1994 [32], adipose tissue began to be considered an active endocrine organ instead of an inert storage organ [33]. After these initial findings, many more inflammatory mediators were shown to be linked to obesity, for example, the anti-inflammatory peptide hormone adiponectin [34], acute-phase proteins such as CRP, and pro-inflammatory cytokines such as IL-6 [35], among more than fifty inflammatory molecules related to adiposity [36,37]. Further research demonstrated that adipose tissue is involved in many physiological and metabolic pathways such as inflammation, insulin sensitivity, and vascular hemostasis [38,39]. It is now well-known that an imbalance of pro-inflammatory and anti-inflammatory adipokines contributes to the development of obesity-related disorders. Anti-inflammatory adipokines dysregulation caused by adipose tissue accumulation also takes part in local and systemic inflammatory responses, thus contributing to the initiation and/or progression of metabolic and cardiovascular disease. Therefore, dysregulation in the synthesis and release of adipokines by adipocytes or infiltrated macrophages in the obese adipose tissue was described and included elevated secretion of pro-inflammatory adipokines (TNF-α, IL-6, leptin, resistin) and reduced secretion of the anti-inflammatory ones (adiponectin) [18]. Furthermore, several researchers also showed that there are metabolic differences between subcutaneous and visceral adipose tissue. This may partly be due to a different release of inflammatory mediators from each specific fat depot [40,41]. 

Interest in this field arose in 2003 when two groups published their observation that inflammatory macrophage presence was significantly higher in obese compared to lean adipose tissue [42,43]. In obesity, a complex immune regulation leads to differentiation, activation, and recruitment of immune cells to key metabolic tissues. An increase in adipose tissue mass causes adipose tissue macrophage accumulation. In turn, macrophages produce pro-inflammatory mediators, including TNF-α and CCL2 (MCP-1) [42,44]. Thus, adipose tissue macrophages play a key role in the development of both local and systemic inflammation and also modulate metabolic features, such as insulin resistance [45]. Due to persistent inflammation of the tissue in obesity, macrophages remain activated and form multinucleate giant cells surrounding individual hypertrophied, dead adipocytes, forming syncytia coined by Cinti and colleagues as crown-like structures (CLS) [46]. CLS can be recognized microscopically when macrophages are immunolabeled with specific antibodies. The majority of macrophages in obese mice and humans are surrounding dead adipocytes, forming CLS, where they may be phagocytizing the lipid remnants of these cells, as an initially protective response that later becomes a persistent site of macrophage activation [46].

In addition, apart from innate immunity cells (macrophages, eosinophils, neutrophils, mast cells), adaptive immune cells such as B and T cells exist in the adipose tissue. Multiple signaling molecules are produced by both innate and adaptive immune cells. The anti-inflammatory cytokines IL-4, IL-13, and IL-10 are mostly produced in lean adipose tissue, whereas the pro-inflammatory cytokines such as INF-γ, IL-12, IL-8, TNF-α, and IL-1β are mainly found in obese adipose tissue. Notably, the numbers and phenotypes of these immune cells are vastly disparate between lean and obese adipose tissue, with cells of obese individuals presenting a more pro-inflammatory profile [31]. Therefore, adipose tissue seems to be an important key for the induction of inflammation, worsened by over-nutrition that causes changes in its cellular composition and production of pro-inflammatory cytokines from infiltrated macrophages and other cells [18].

### 1.4. Immunometabolism: A Recent Concept

Despite the extensive literature on the topic, very little is still known about whether and how the immune system contributes to worsened metabolic conditions. It seems that the organism must readjust its set point referred to blood levels of glucose and energy balance to achieve homeostasis. In spite of the marked reduction in sensitivity to insulin, as mentioned above, the organism tends to reduce its energy expenditure in order to preserve energy storage. One way in which this aim is achieved in adipose tissue is through the development of resistance to lipolytic or thermogenic signals, such as catecholamines or leptin. Simultaneously, continued unresolved inflammation, angiogenesis and adipose tissue expansion eventually lead to fibrosis, which is linked to metabolic inflexibility, dysregulation of metabolic pathways, and adipocyte death, resulting in the adverse effect of chronic inflammation mentioned above [28].

In this context, in the last years, there have been significant advancements in this line of research, constituting a new field, termed “immunometabolism”, that merges immunology and metabolism [47]. Immunometabolism comprises two aspects: one is the effect of inflammation and immune responses on the control of systemic metabolism, and the other focuses on metabolism within immune cells and changes in the function of immune cells depending on metabolic changes in the organism, changing their metabolic profile to obtain the necessary energy for defensive processes or homeostatic immunoregulation. Clearly, metabolic changes in immune cells have important effects on their function, which in turn have important implications in cardiometabolic diseases and cancer.

The close link between immunity and nutrition is evidenced by the fact that the immune system is one of the most energy-consuming systems in the body; therefore, a nutrient imbalance can have a strong impact [48]. Immune activation is associated with energetic and biosynthetic demands, and immune cells must engage in metabolic reprogramming to generate sufficient energy to fuel these demands [49]. Although fat produces more energy than glucose does (and glucose produces more energy in aerobic metabolism in the presence of oxygen than without oxygen), energy production is faster using glucose as an energy substrate (to a greater extent when burned anaerobically). Therefore, in the event of an infection, the acute immune response must be fast using glucose as an energy substrate. In general, inflammatory activation of various immune cells is associated with a marked shift toward glycolytic metabolism and lower reliance on fat oxidation. The increase in glycolysis triggers HIF1α-mediated gene transcription and cytokine production [50]. However, in a basal situation or in long-term regenerative processes after acute inflammatory responses, immune cells can use fat as an energy substrate. This ability to respond or adapt to changes in metabolic demand was termed metabolic flexibility. Thus, immune cells metabolic profile may be modulated in different healthy and disease conditions by manipulating nutrient availability and other interventions that restore dysregulated immune response [51,52].

In this context, there are different metabolic pathways that can modify immune cell behavior, particularly glycolysis, pentose phosphate pathway, fatty acid oxidation, fatty acid synthesis, Krebs cycle, and amino acid metabolism, and the metabolites produced in these different pathways can determine and shape the immune response. In the absence of infection, the energy demand of immune cells is low as they do not require activation and/or proliferation; therefore, oxidation in the Krebs cycle may be sufficient. However, upon pathogen attack and in the presence of infection, fermentative glycolysis and the pentose phosphate pathway are required to provide more anabolic material to allow immune cells to activate and proliferate [51,53]. In fact, during an infectious process, the great increase in energy demand by the immune system causes immune cells to stop using the Krebs cycle to ferment glucose and thus obtain anabolites to proliferate through less efficient but faster metabolic pathways [54].

For instance, in recent years, there has been growing evidence that immunometabolic changes in obesity may promote effector T-cell differentiation and inflammatory cytokine production, increasing the risk of obesity-associated metabolic diseases. Interactions between fat metabolism and immunity were evidenced by studies showing that dietary lipid manipulation affects fatty acid composition of phospholipids from splenocyte membranes, which can influence lymphocyte functions [48]. Furthermore, polyunsaturated fatty acids induce immunosuppressive effects caused by an increase in linoleic acid or a decrease in oleic acid, modifying many components of plasma membrane-associated events involved in lymphocyte activation [55]. Activation of anaerobic glycolysis is a common feature of inflammatory activation of T cells [56,57], dendritic cells [58], and macrophages [59]. However, further studies are required to fully understand the extent to which adaptive immunity contributes to insulin sensitivity [30].

Another example of the crosstalk between nutrients and inflammation is that circulating lipids have a role in modulating insulin sensitivity. Toll-like receptor 4 (TLR4), a component of the innate response, is a receptor for saturated and polyunsaturated fatty acids, suggesting that lipids may have immunomodulatory functions [30]. Thus, TLR4 can induce the release of pro-inflammatory cytokines by macrophages, constituting a molecular link among nutrition, lipids, and inflammation. In this way, the innate immune system participates in the regulation of energy balance and insulin resistance in response to changes in the nutritional environment [60].

Moreover, it is important to acknowledge the role of certain inflammatory mediators, released mainly by immune cells in metabolic homeostasis. TNF-α is one of the main pro-inflammatory mediators that contribute to the development of insulin resistance in adipocytes and peripheral tissues by inducing low-grade inflammation in those tissues. TNF-α reduces the expression of GLUT4 and promotes serine phosphorylation of IRS-1, which are important molecules for insulin signaling [61]. In addition, the multifunctional cytokine IL-6 is involved in the regulation of metabolism, stress, and the immune system but is still the subject of controversies regarding its physiological functions [18]. Circulating IL-6 contributes to the development of atherosclerosis and insulin resistance, the latter being caused by IL-6-induced impairment of the phosphorylation of insulin receptor and insulin receptor substrate-1 by inducing the expression of SOCS-3, a potential inhibitor of insulin signaling [62]. Therefore, IL-6 reduces hepatic insulin sensitivity and glucose uptake by adipocytes and causes elevated plasma insulin levels, hyperglycemia, and hyperlipidemia. However, other studies suggest that IL-6 has a lipolytic role, with its participation in lipolysis and fat oxidation [63]. Several other inflammatory cytokines and mediators such as interleukin 1β (IL-1β) have also been implicated in the pathogenesis of insulin action or secretion [64]. IL-1β is one of the major pro-inflammatory cytokines produced by macrophages, and it leads to the activation of NF-κB pathways and the generation of other inflammatory molecules, such as TNF-α and IL-1β itself, thus initiating a positive feedback loop [15]. Thus, all of these pro-inflammatory cytokines act in an autocrine and paracrine manner to promote insulin resistance by interfering with insulin signaling in peripheral tissues [65].

Taking all of this into account, it seems clear that different tissues such as white, brown, and beige adipose tissue, muscle, liver, and the central nervous system play a role in obesity-associated metabolic disease. Particularly, white adipose tissue is crucial in obesity since prolonged overnutrition leads to an accumulation of adiposity and metabolic abnormalities that negatively impacts immune function and host defense [66]. As it was demonstrated, the metabolism of immune cells plays a critical role in generating different immune responses, and in the context of immunometabolism, macrophages are one of the most relevant immune cells. Metabolic flexibility in the utilization of fat or glucose is very important in macrophages to achieve good immune homeostasis, particularly in the context of obesity and white adipose tissue inflammation. M1 macrophages need to use glucose to develop a fast inflammatory and microbicidal response to pathogen challenge, whereas M2 macrophages will primarily use fat to develop their anti-inflammatory and tissue regeneration function, all of this contributing to optimal immunoregulation or inflammatory homeostasis. 

Several genetic and pharmacologic strategies aimed at reducing the inflammatory state or adipose tissue macrophage content in obese rodents can modulate local inflammation and improve insulin resistance [67]. Nevertheless, many of the metabolic factors that regulate the immune response and the accumulation of macrophages and other immune cells in the obese adipose tissue remain poorly defined [24].

## 2. Obesity, Immunometabolism, and Macrophages

Adipose tissue inflammation in mice fed a high-fat diet contributes to systemic insulin resistance and glucose intolerance [68]. In obesity, different types of innate and adaptive immune cells accumulate in adipose tissue with increased adipocyte chemokine production [69,70]. Thus, apart from leukocytes, multiple cell types can contribute to the inflammatory environment, including adipocytes and stromal cells [42,43]. However, macrophages are the most abundant cells in obese adipose tissue [71] and represent the major source of pro-inflammatory cytokines, thus inducing systemic insulin resistance [43]. It is important to note that innate immune cells also have important functions in healthy tissue physiology. Indeed, immune cells are also present in healthy adipose tissue contributing to tissue homeostasis and playing a critical role in the healthy expansion and remodeling of the tissue during weight gain [72,73].

Resident and infiltrating macrophages are responsible for different types of functions, both in health situations and in response to damage and infection. Macrophages perform phagocytosis of an array of infectious microorganisms, phagocytose tissue debris, apoptotic parenchymal cells and neutrophils, repair wounded tissue, and act as a bridge between innate and adaptive immunity. In accordance with these diverse functions, macrophages show different phenotypes [74]. In macrophages, arginine is a precursor for two important amino acid metabolic pathways: it is either metabolized by inducible nitric oxide synthase (iNOS) to nitric oxide (NO) and citrulline (classical pathway) or hydrolyzed by arginase to ornithine and urea (alternative pathway). The existence of these two metabolic pathways has crucial implications for their phenotype and function, and therefore, for the type and outcome of immune responses in which these cells are involved [75]. M1 phenotype refers to the “classically-activated” macrophage that appears during cell-mediated immune responses and is characterized by a pro-inflammatory phenotype. Interferon-γ (IFN-γ) and lipopolysaccharide (LPS) prime macrophages to acquire the microbicidal and pro-inflammatory properties that characterize this phenotype, and they are the first line of defense against intracellular pathogens stimulating Th1 polarization of CD4+ lymphocytes. They are characterized by the expression of CD11c and inducible nitric oxide synthase (iNOS) and are more prevalent than macrophages with M2 anti-inflammatory phenotype in obesity. On the other hand, M2 or alternatively activated macrophages are the counterparts of Th2 lymphocytes and are characterized by the expression of CD206 and type-1 arginase (ARG1). M1 macrophages are associated with the production of pro-inflammatory mediators (such as IL-1β, IL-6, TNF-α), high microbicidal activity, and cellular immunity, while M2 macrophages are associated with the release of anti-inflammatory cytokines such as IL-10 (which contributes to the insulin-sensitive phenotype), tissue repair, remodeling processes, and humoral immunity [18,76,77]. Macrophages are reported to potentially switch from one phenotype or state to another depending upon environmental stimuli [78]. The predominant presence of tissue-resident macrophages with an anti-inflammatory phenotype, that is, polarized towards repair and maintenance functions, in lean adipose tissue suggests a physiological role for these cells in homeostasis. Moreover, it indicates that during metabolic stress and obesity, they are reprogrammed into a pro-inflammatory phenotype, leading to functional deterioration [30].

Most adipose tissue macrophages express the surface markers CD11b and F4/80. In obesity, the majority of recruited adipose tissue macrophages are also CD11c+, with increased expression of pro-inflammatory genes, that is, macrophages with a pro-inflammatory phenotype [71,79]. People who are lean, physically active, and follow a healthy diet maintain an “anti-inflammatory phenotype of adipose tissue” (small adipocyte size and the presence of M2 macrophages). On the other hand, physical inactivity and unhealthy diets provoke a positive energy balance, which leads to an increase in visceral fat that induces the development of “inflamed adipose tissue”, characterized by infiltrated M1 macrophages, correlating with insulin resistance in both mice and humans [80,81]. Recruitment of circulating bone marrow-derived monocytes as well as proliferation contributes to CD11c+ adipose tissue macrophage accumulation and maintenance [82,83,84], and adipose tissue T-cell–dependent signals were also demonstrated to promote CD11c+ adipose tissue macrophage accumulation and inflammation with obesity [85]. The metabolic factors that modulate the immune response in obesity and the aggregation of macrophages and other immune cells in adipose tissue are not still well known [42,86,87]. Cinti and colleagues suggested that adipocyte necrotic-like death, which they hypothesize is driven by hypertrophy and accelerated by obesity, is the primary stimulus that regulates adipose tissue macrophage accumulation [46]. Indeed, it was also demonstrated that massive adipocyte apoptosis leads to the rapid accumulation of adipose tissue macrophages [88]. In this way, one of the initiating causes of adipocyte pro-inflammatory signaling is hypoxia within white adipose tissue, which occurs very early in the onset of obesity [68,89]. Free fatty acids are increased in obesity and are implicated in insulin resistance and induction of inflammatory signaling in adipose, liver, muscle, and pancreas, where they serve as ligands for the TLR4 complex, activate a classical inflammatory response and drive the accumulation of adipose tissue macrophages [77]. In diet-induced obesity, the accumulation of triglycerides per se is a relatively favorable process. However, adipocytes’ enzymatic and structural capacity to store fatty acids is eventually surpassed by an ongoing excess of calories [90]. Moreover, the response of the adipose vasculature and stroma to adipose expansion is often insufficient, driving to hypoxia and overcrowding. The failure to store excess fatty acids causes an increase in the concentration of free fatty acids and lipids synthesized from fatty acids to a harmful level. This event perturbs adipocyte integrity and function and may cause apoptosis and necrosis [91,92]. In this context, an initiating mechanism of hypoxia involves uncoupling of mitochondria oxidative metabolism as a result of elevated free fatty acids [89]. This leads to increased oxygen consumption within adipocytes, creating relative adipocyte hypoxia [89,93]. 

Increases in adipose tissue mass and adipocyte volume have other metabolic consequences reflected in a reduced mitochondrial function, increased endoplasmic reticulum stress, impaired insulin signaling, and higher rates of basal lipolysis [94,95,96]. The function of adipose tissue macrophages in the obese state was suggested to be phagocytosis of excess lipid since, with increasing adiposity, they form multinucleated syncytia that contain large lipid droplets [46,97]. This close link between adipocyte size, macrophage accumulation, and lipid uptake proposes that excess lipids may be crucial for adipose tissue macrophage accumulation. In this context, it was hypothesized that the immune system and macrophages respond directly to alterations in metabolic function, and specifically, that obesity-induced increases in basal lipolysis [94] can increase local extracellular lipid concentrations and drive adipose tissue macrophage accumulation. If increased lipolysis drives adipose tissue macrophage accumulation, then altering lipolysis should modulate adipose tissue macrophage accumulation in the same way. These findings suggest that adipose tissue macrophages may play a role in tempering extracellular boosting in free fatty acids concentrations during periods of high lipolysis and may thus protect local adipocyte function. In lean individuals, adipocytes store little lipid, basal lipolysis is limited, and adipose tissue macrophages are few. In obesity, excess accumulation of lipid by large adipocytes increases basal lipolysis, and therefore, also causes a clear release of free fatty acids. Macrophages are recruited to the adipose tissue, accumulating rapidly, and chronic stimulation of these cells contributes to local inflammation and altered metabolic function [24]. Increased release of TNF-α (together with MCP-1 and IL-8) recruits more macrophages and T lymphocytes, further causing a situation of hypoxia that increases local inflammation and fibrosis in adipose tissue [98]. Once recruited, adipose tissue macrophages phagocytose excess lipid, possibly reducing adipocyte stress. During weight loss, an increase in demand lipolysis raises local free fatty acids concentrations and thus adipose tissue macrophage recruitment. However, progressively, as triglyceride stores decrease and basal lipolysis decreases, adipose tissue macrophage content is reduced [24]. Furthermore, one of the most well-known metabolic changes in the inflammatory activation of macrophages is the activation of anaerobic glycolysis [59], allowing a survival advantage in hypoxic environments such as adipose tissue from obese individuals [99]; together with the accumulation of Kreb’s cycle intermediates that are important for the production of inflammatory cytokines [100]. In macrophage polarization, fatty acid oxidation plays a key role in transcriptional regulation. M1 macrophage polarization relies on glycolytic activity, whereas differentiation into M2 macrophages (activated by IL-4) requires fatty acid oxidation [101]. Nevertheless, one has to be careful when defining glycolysis as pro-inflammatory and fatty acid oxidation as anti-inflammatory. This can be an oversimplification due to the implication of both metabolic routes in pro-inflammatory and anti-inflammatory activity [102].

In this context, it is important to note that, as mentioned above, macrophages can contribute to the regulation of energy balance and insulin resistance in response to changes in the nutritional environment since their receptor TLR4 can recognize fatty acids and lead to the release of pro-inflammatory cytokines by these cells [60]. More recent studies have carefully examined the activation and functional impact of adipose tissue macrophages. For example, Kratz and colleagues [103] demonstrated that some classical macrophage markers of inflammation are not induced in response to metabolic stimuli, which suggests that alternative activation pathways, including fatty acid-driven PPARγ signaling, may underlie metaflammatory phenotypes; Shan et al. [104] showed that chronic endoplasmic reticulum stress in obesity might lead to inflammatory polarization in adipose tissue macrophages. 

TNF-α is also implicated in lipolysis and insulin resistance in the inflamed adipose tissue. Most of the TNF-α in inflamed adipose tissue is produced in adipocytes (where most of its mRNA is located), so there seems to be a direct relationship between the amount of fat and the release of this inflammatory cytokine. TNFα released into the tissue is recognized by its receptors TNF-R1 to stimulate lipolysis and apoptotic processes and TNF-R2, involved in insulin resistance through inhibition of insulin signaling [105,106,107]. The other cytokine that contributes greatly to low-grade inflammation in obesity is IL-6. Although 30% of total IL-6 production, partly stimulated by TNF-α, appears to come from adipose tissue, adipocytes contribute only up to 10% as the rest is produced by macrophages infiltrating adipose tissue [105,108], with a shift towards their M1 inflammatory phenotype as fat depots increase, producing more TNF-α, IL-6, and IL-1β and contributing to insulin resistance [109]. Localized visceral fat contributes more to chronic low-grade inflammation than the overall accumulation of fat in the body, since the increase in the amount of visceral fat, particularly in the abdomen and liver, implies a greater infiltration of macrophages and levels of inflammatory cytokines and the decrease in visceral fat decreases inflammatory markers and can lead to weight loss [108].

Apart from adipose tissue macrophages, some studies focused on non-infiltrated peritoneal macrophages. Research from our group showed that in obese Zucker rats, peritoneal macrophages present a local dysregulation in the constitutive or spontaneous release of pro-inflammatory cytokines such as IL-1β, INF-γ, IL-6, and TNF-α, contributing to the low-grade systemic pro-inflammatory state in obesity. The release of those pro-inflammatory cytokines is also impaired in response to antigenic (LPS) stimulus, thus potentially increasing susceptibility to infection [11,110]. Peritoneal macrophages from obese mice show different inflammatory responses to adrenergic stimulation, with more drastic anti-inflammatory effects than in lean mice [111]. Moreover, monocytes, which are macrophage precursors, also present a differential inflammatory response to adrenergic stimulation in obesity compared to lean individuals, with an anti-inflammatory effect that occurs particularly in obese individuals [20,111]. Obese mice also show a lower monocyte-mediated innate response [10]. 

## 3. Weight Loss, Inflammation, and Macrophages

Bearing all of the above in mind, it seems clear that evidence strongly supports the relevance of immunometabolism in health and disease, with potential clinical value and highly promising new areas and mechanisms for intervention that are being developed, particularly in the field of obesity and its metabolic complications, such as type 2 diabetes.

Weight loss is the main objective and primary recommendation for the management of obesity and obesity-related comorbidities. In order to achieve weight reduction, various intervention strategies such as anti-obesity drugs, bariatric surgery, and lifestyle interventions are taken into consideration [112]. Among these, decreasing caloric intake (dietary interventions such as caloric restriction or abandonment of a high-fat diet) and following a physical exercise program are by far the most accepted methods to promote weight loss. In the present review, we focused on dietary interventions since they are the first lifestyle change that patients address, and they represent fundamental non-pharmacological and non-surgical strategies in this condition, with a major impact on health parameters, and can even interfere and determine the outcomes of other concomitant strategies such as exercise.

Those interventions can help improve metabolic dysfunction and reduce markers of inflammation within adipose tissue [113,114]. The reduction in disease risk in response to weight loss could be a result of an improved inflammatory profile, which involves a decrease in pro-inflammatory mediators and an increase in anti-inflammatory ones [115]. Weight loss has been shown to improve inflammation in terms of several inflammatory markers associated with obesity, specifically decreases in pro-inflammatory biomarkers (CRP, TNF-α, IL-6, and leptin) and increases in the anti-inflammatory biomarker adiponectin [116]. However, in this context, it is still necessary to clarify the clinical implications of the alterations induced by obesity related to immunity and by some of those interventions [5]. 

Beneficial metabolic effects of weight loss have been extensively described, such as the reversibility of the atherogenic lipoprotein profile by lowering triglycerides, improving the insulin to glucose ratio, and increasing HDL cholesterol [117,118,119]. Moderate weight loss improves insulin sensitivity and many of the frequent medical complications associated with obesity, such as type 2 diabetes [120,121]. Although a 10–20% reduction in weight can have significant metabolic improvements with improved glucose and insulin levels [122], effects on the chronic inflammatory state of obesity have been less thoroughly investigated. This practice, through lifestyle or surgical interventions, can decrease adipose tissue macrophage numbers, reduce inflammation, and improve insulin sensitivity [24,123]. Moreover, weight loss is now accepted to be associated with decreased concentrations of inflammation-related products in circulation [124,125,126]. 

As referred to before, the presence of macrophages can contribute to the increased expression of inflammatory genes in obese subjects. Thus, non-pharmacologic and pharmacologic manipulations that reduce adipose tissue macrophage content or modify their inflammatory state in obese animals modulate local inflammation and are associated with a reduction in insulin resistance [24]. It was described that caloric restriction of high-fat diet-fed mice led to an initial increase in adipose tissue macrophage recruitment, while adipose tissue macrophage content was shown to be reduced following an extended period of weight loss. The peak in adipose tissue macrophage number coincided with the peak in the circulating concentrations of free fatty acids and adipose tissue lipolysis, suggesting that lipolysis drives adipose tissue macrophage accumulation [24]. Certainly, lipolysis induced by fasting or pharmacological approaches rapidly increased adipose tissue macrophage accumulation, chemoattractant activity, and lipid uptake by adipose tissue macrophages. Conversely, dietary and genetic manipulations that reduced lipolysis declined adipose tissue macrophage accumulation. Depletion of macrophages in adipose tissue cultures increased expression of adipose triglyceride lipase and genes regulated by free fatty acids and increased lipolysis. These data suggest that local lipid fluxes are central regulators of adipose tissue macrophage recruitment and that once recruited, adipose tissue macrophages form lipid-laden macrophages that can buffer local increases in lipid concentration, according to Kosteli and colleagues [24]. In conclusion, macrophage and fat cell gene expression in adipose tissue can be differentially regulated during the calorie restriction and weight maintenance within a weight loss program; thus, diet-induced weight loss was suggested to be associated with a reduction in systemic inflammation and specific metabolic adaptations, indicating, again, the interaction between nutrition, the immune system, and metabolism [127,128]. 

Therefore, there is much evidence that weight loss has an impact on the inflammatory profile of obese and former obese subjects; however, most results are controversial and contradictory, which can be due to the different methodological approaches and dietary interventions analyzed. It was shown that weight loss dramatically reduced inflammation in the liver, skeletal muscle, and heart, but not in adipose tissue. The application of a low-fat diet mediating weight loss reduced lipid levels and improved insulin sensitivity selectively in the liver, also enhancing cardiac glucose metabolism [129]. Schmitz and colleagues demonstrated that weight loss resolved pro-inflammatory gene expression exclusively in the liver, whereas visceral adipose tissue displayed no significant improvement of metabolic and inflammatory markers in comparison to obese mice [130]. In a study carried out in obese women, weight loss reduced colorectal mucosal inflammation by decreasing inflammatory cytokines and cells and by down-regulating inflammatory and cancer gene pathways [131]. Another study in a group of obese women examined the effect of a 6-month dietary intervention consisting of two periods: 4 weeks of very-low-calorie diet followed by weight stabilization (2 months of low-calorie diet and 3–4 months of weight maintenance diet). After the short-term severe calorie restriction, adipose tissue macrophages content was not modified; however, it considerably decreased in the weight stabilization period. This adipose tissue macrophage reduction was not accompanied by a variation in macrophage phenotype [132]. It was also demonstrated that low-grade systemic inflammation in post-obese men (individuals who are no longer obese) with a long-term sustained weight loss was similar to non-obese controls despite a higher subcutaneous adipose tissue CD68+ macrophage content [133]. In another study, mice were divided into three dietary groups: high-fat diet, low-fat diet, and restriction to 70% of the high-fat diet. Both groups fed with a low-fat diet and 70% of the high-fat diet showed beneficial effects in terms of physiological and metabolic parameters. Macrophage infiltration in white adipose tissue was reduced by both interventions, even more remarkably in a restriction to 70% of the high-fat diet. Activation of mitochondrial carbohydrate and fat metabolism was increased in mice with restriction to 70% of the high-fat diet. Thus, it seems that a 30% restriction of a high-fat diet induced more pronounced effects than a change to a low-fat diet, in particular, regarding adipose tissue inflammation and expression of mitochondrial carbohydrate metabolism genes [134]. 

Nevertheless, as we mentioned, some of those results seem to be controversial, as there are some studies proving that weight loss is associated with the persistent expression of inflammatory cytokines such as interleukin IL-6, IL-1β, and TNF-α in adipose tissue [129,135], which means adipose tissue inflammation and insulin sensitivity is not fully resolved after weight loss [114]. Blaszczak and colleagues observed that mice ingesting 3 months of high-fat diet followed by 3 months of chow-diet remained more insulin resistant and glucose-intolerant than chow-fed animals and also that adipocytes, adipose tissue regulatory T cells, CD8+ T cells, type 2 innate lymphoid cells, and M1-like macrophages all failed to normalize after weight loss [136]. In a study performed by Zamarron and colleagues, adipose tissue leukocytes in mice were examined after withdrawal of a high-fat diet. After 8 weeks of weight loss through the abandonment of the high-fat diet, the mice’s weight and glucose tolerance were similar to age-matched lean controls, but they showed abnormal insulin tolerance. Moreover, adipose tissue macrophages (CD45+, CD64+) were reduced during the weight loss process but remained significantly high compared with normal diet-fed mice, despite similar adipose tissue weights. Thus, total and pro-inflammatory CD11c+ adipose tissue macrophage content remained elevated in mice of the weight loss group. This adipose tissue macrophage content in formerly obese mice showed a pro-inflammatory profile, including elevated expression of interferon-γ, TNF-α, and interleukin-1β. Authors concluded that formerly obese mice have long-term alterations in adipose tissue macrophage composition, so weight loss does not completely resolve obesity-induced adipose tissue macrophages activation, which may contribute to the persistent adipose tissue damage and reduced insulin sensitivity present in formerly obese mice [114]. 

Differential results can be explained by several factors, since effects may be proportional to the degree of weight loss, as weight loss around 15%, but not 5%, has demonstrated a result of a decrease in adipose tissue inflammation. Furthermore, the increase and persistence of liver and adipose tissue inflammation were also demonstrated to be gender-dependent, as male mice showed a persistent inflammation even after weight loss despite metabolic improvement [137]. Therefore, bearing in mind the controversy and dissimilar results in the studies examining the use of low-calorie diets or restriction of high-fat diets in order to achieve weight loss, it is important to consider other diets, nutritional strategies, or the use of functional foods. For instance, ketogenic diets were proposed to be useful to control inflammation. The ketogenic diet, a diet high in fat and low in carbohydrates (thus utilizing fat and lipid metabolism as the main energy source), has demonstrated to lead to significant weight loss and has also been associated with a decrease in systemic inflammation, reduced insulin resistance and fasting blood glucose levels, and improved lipid profile and oxidative stress [138,139]. However, other studies show conflicting results pointing to increased cholesterol and inflammatory markers, decreased triglycerides, and decreased insulin-mediated antilipolysis [140]. Again, we find differing results and a limited number of studies, which together with long-term severe adverse effects detected after this intervention [139], clearly reveal that further research is needed in this area before proposing its implementation as an anti-inflammatory strategy in obesity.

## 4. Conclusions

Besides outcomes regarding the inflammatory response, since obese individuals present higher susceptibility to infection mainly due to lower phagocytic and microbicide capacity of innate cells (monocytes/macrophages) [10,18], among other alterations, it would be crucial to investigate the effects of weight loss through dietary interventions (fundamental abandonment of a high-fat diet) on the innate immune response. To the best of our knowledge, this aspect has not been elucidated yet, and it would be very relevant to examine if weight loss based on low-calorie diets or restriction of the high-fat diet has any effect on this aspect of the immune system. Since potential anti-inflammatory effects of weight-loss strategies (drugs, dietary interventions, exercise) could compromise the immune system’s effectiveness against pathogen challenge, achieving bioregulation of the inflammatory/innate responses are key to determining if the intervention is successful and appropriate. In this way, the “bioregulatory effect of the inflammatory/innate responses” (a term originally coined in the context of exercise and other anti-inflammatory strategies such as hyperthermia by balneotherapy) [22,141] is the one that reduces or prevents an excessive and sterile inflammatory response but also preserves or stimulates the innate response. Good transitions between pro- and anti-inflammatory macrophages should also be present. All of this must be adapted to each individual’s inflammatory set-point in low-grade inflammation-related diseases, particularly in obesity [10,21,22]. Nevertheless, from this review, it can be concluded that most of the recent studies investigating the effects of weight loss through dietary interventions without physical exercise do not seem to point to an improvement of the inflammatory state, especially in the adipose tissue, even when metabolic improvements take place. This is a very important new approach to evaluate good bioregulatory effects of the innate/inflammatory immune response in the different weight-loss strategies in the absence of exercise, which is particularly relevant in the context of viral infectious diseases in which obese individuals constitute the main risk population for susceptibility and worse prognosis, as it has clearly been reported in COVID-19.
